# Neurological Pupillary Index and Disposition at Hospital Discharge following ICU Admission for Acute Brain Injury

**DOI:** 10.3390/jcm12113806

**Published:** 2023-06-01

**Authors:** Abhijit V. Lele, Sarah Wahlster, Sunita Khadka, Andrew M. Walters, Christine T. Fong, Patricia A. Blissitt, Sarah L. Livesay, Gemi E. Jannotta, Bernice G. Gulek, Vasisht Srinivasan, Kathryn Rosenblatt, Michael J. Souter, Monica S. Vavilala

**Affiliations:** 1 Neurocritical Care Service, Harborview Medical Center, University of Washington, Seattle, WA 98195, USA; wahlster@uw.edu (S.W.); khadkas@uw.edu (S.K.); slivesay@uw.edu (S.L.L.); jannotta@uw.edu (G.E.J.); gulekb@uw.edu (B.G.G.); vasishts@uw.edu (V.S.); msouter@uw.edu (M.J.S.); 2 Department of Anesthesiology and Pain Medicine, Harborview Medical Center, University of Washington, Seattle, WA 98195, USA; walters3@uw.edu (A.M.W.); cfong@uw.edu (C.T.F.); vavilala@uw.edu (M.S.V.); 3 Department of Neurological Surgery, Harborview Medical Center, University of Washington, Seattle, WA 98195, USA; 4 Harborview Injury Prevention and Research Center, Harborview Medical Center, University of Washington, Seattle, WA 98195, USA; 5 Department of Neurology, Harborview Medical Center, University of Washington, Seattle, WA 98195, USA; 6 Department of Nursing, Harborview Medical Center, University of Washington, Seattle, WA 98195, USA; pbliss@uw.edu; 7 Department of Emergency Medicine, Harborview Medical Center, University of Washington, Seattle, WA 98195, USA; 8 Department of Anesthesiology and Critical Care Medicine, Johns Hopkins University School of Medicine, Baltimore, MD 21205, USA; krosenb3@jhmi.edu

**Keywords:** pupillometer, NPi, outcomes, neurocritical care, neurological pupillary index, automated pupillometry

## Abstract

We examined the associations between the Neurological Pupillary Index (NPi) and disposition at hospital discharge in patients admitted to the neurocritical care unit with acute brain injury (ABI) due to acute ischemic stroke (AIS), spontaneous intracerebral hemorrhage (sICH), aneurysmal subarachnoid hemorrhage (SAH), and traumatic brain injury (TBI). The primary outcome was discharge disposition (home/acute rehabilitation vs. death/hospice/skilled nursing facility). Secondary outcomes were tracheostomy tube placement and transition to comfort measures. Among 2258 patients who received serial NPi assessments within the first seven days of ICU admission, 47.7% (n = 1078) demonstrated NPi ≥ 3 on initial and final assessments, 30.1% (n = 680) had initial NPI < 3 that never improved, 19% (n = 430) had initial NPi ≥ 3, which subsequently worsened to <3 and never recovered, and 3.1% (n = 70) had initial NPi < 3, which improved to ≥3. After adjusting for age, sex, admitting diagnosis, admission Glasgow Coma Scale score, craniotomy/craniectomy, and hyperosmolar therapy, NPi values that remained <3 or worsened from ≥3 to <3 were associated with poor outcomes (adjusted odds ratio, aOR 2.58, 95% CI [2.03; 3.28]), placement of a tracheostomy tube (aOR 1.58, 95% CI [1.13; 2.22]), and transition to comfort measures only (aOR 2.12, 95% CI [1.67; 2.70]). Our study suggests that serial NPi assessments during the first seven days of ICU admission may be helpful in predicting outcomes and guiding clinical decision-making in patients with ABI. Further studies are needed to evaluate the potential benefit of interventions to improve NPi trends in this population.

## 1. Introduction

Acute brain injuries (ABIs) are devastating events that require close monitoring in neurocritical care units (NCCUs) due to the risk of imminent and life-threatening neurological deterioration [1,2]. Pupillary exams are an essential component of neurological monitoring that provide valuable information about intracranial pressure changes and cranial nerve function and guide medical and surgical interventions. Automated portable pupillometers (APMs) are hand-held infrared systems that automatically track and analyze pupil dynamics over 3 s and have been widely used [3,4] in patients with ABI requiring neurological/neurosurgical care. APM measures the Neurological Pupillary Index (NPi) (NPi, NeurOptics, Irvine, CA, USA) using a light flash of fixed intensity and duration, which stimulates the pupil light constriction reflex and serves as a quantitative surrogate of pupillary reactivity.

Several neuronal and mechanical mechanisms influence the pupillary light reflex. Pupil size, constriction velocity, dilation velocity, and latency are all important variables measured using a pupillometer and included in the NPi algorithm [5]. Each variable measured by the pupillometer is compared with the mean of a reference distribution of healthy subjects for the same variable by standardizing the difference with the corresponding standard deviation (z-score). All z-scores are combined and fall within a scale from 0 to 5, reflecting a multidimensional normative model. Pupil reactivity falls within the normative pupil behavior distribution (i.e., pupillary reaction to light is “normal” or “brisk”) with an NPi score ≥ 3, with an NPi of 5 indicating more “brisk” than an NPi of 3 [6]. An NPi score < 3 indicates an abnormal pupillary light reflex (i.e., “sluggish” or weaker than a normal response), with a value of 1 being more abnormal than a value of 3. An NPi of 0 indicates a non-reacting pupil, which may require urgent intervention.

Studies have shown that the NPi value provides critical information for predicting outcomes in patients with ABI. NPi values < 3 within the first three days of hospitalization have been identified as an early predictor of unfavorable 6-month outcomes in ABI [7]. Furthermore, NPi values < 2.8 have been associated with the risk of neurological deterioration after large hemispheric strokes [8], while patients with cerebral herniation may exhibit NPi values closer to 0 [9]. NPi is also an adjunct tool for neurological prognostication following out-of-hospital cardiac arrest. Combining NPI at 0 h after the return of spontaneous circulation with clinical predictors improves prognostic values from an AUC of 0.92 with NPi alone to an AUC of 0.95–0.96. NPi may predict unfavorable outcomes as early as day one after cardiac arrest, demonstrating higher specificity than standard manual pupillary examinations [10]. Adding NPi to somatosensory evoked potentials can also increase the sensitivity of outcome predictions after cardiac arrest [11].

While the clinical course of patients with ABI is often highly dynamic in the early days after hospitalization, no clinical studies have investigated the association between NPi trends and clinical outcomes at the time of hospital discharge. In this large, single-center retrospective observational study, we describe trends in NPi over time and examine their association with discharge outcomes in patients with ABIs. Our analysis can provide additional information about the prognostic value of APM, which may guide the development of targeted interventions to improve outcomes for patients with ABI.

## 2. Materials and Methods

### 2.1. Institutional Review Board Approval

The University of Washington Institutional Review Board approved this study (STUDY00009382) on 1 March 2022, using a waiver of informed consent.

### 2.2. Study Setting and Patient Selection

This retrospective cohort study was conducted at Harborview Medical Center, a 413-bed Level 1 trauma center, comprehensive stroke center, and high-volume aneurysmal subarachnoid referral center. All adult patients (age ≥ 18 years) admitted to the NCCU between 1 January 2014 and 1 February 2022 with acute ischemic stroke, spontaneous intracerebral hemorrhage, subarachnoid hemorrhage, and traumatic brain injury who had APM recordings were included. Patients younger than 18 years and patients without APM recordings were excluded. 

### 2.3. Data Collection

Patient characteristics and brain injury severity data collected from the electronic medical records included patient age, sex, admission diagnosis, Glasgow Coma Scale (GCS) at ICU admission and hospital discharge, mechanical ventilation at any point, and intracranial pressure monitoring, electroencephalography, craniotomy/craniectomy. Based on the GCS at ICU admission, patients were categorized into three groups: mild (GCS 13–15), moderate (GCS 9–12), and severe (GCS 3–8). 

APM data extracted from the EMR were the lowest NPi recorded between the left-sided and right-sided pupils from every assessment recorded during the first seven days of the ICU admission. 

We categorized patients into four groups based on NPi trajectories, using their first NPi assessment and last assessment performed during their ICU stay:

1. Normal and stable = initial NPi ≥ 3 or greater that remained ≥3;
2. Improved = initial NPi < 3, but subsequently improved to ≥3;
3. Abnormal and stable = initial NPi < 3, and remained <3;
4. Worsened = initial NPi ≥ 3, which worsened to <3 and did not recover.

### 2.4. Outcomes

The primary outcome was the discharge disposition dichotomized as good (discharge to home/rehabilitation facility) vs. poor (death/hospice/skilled nursing facility) outcomes.

Secondary outcomes were tracheostomy tube placement and transition to comfort measures only (CMO). Acute rehabilitation or home discharge generally corresponds to a modified Rankin score or mRS of 0–3, while skilled nursing facility/hospice/death corresponds to a modified Rankin score of 4–6. Understanding the functional criteria for acute rehab (≥3 h of sustained participation in activity per day) or home (mostly independent) is important as we make this association.

### 2.5. Statistical Analysis

A descriptive analysis was performed to describe cohort characteristics. Categorical data were presented as counts and percentages. Shapiro–Wilk tests were performed to test the normality of continuous variables such as age, NPi, ICU, and hospital LOS. Data were presented as the mean ± standard deviation (SD) or median (interquartile range, or IQR). One-way analysis of variance testing was used to examine differences in intensive care and hospital length of stay by the NPi trajectory category. The multivariable logistics regression model tested the association between the NPi trajectory and outcomes (model adjusted for age, sex, admission diagnosis, craniotomy/craniectomy, hyperosmolar therapy, and GCS). A Hosmer–Lemeshow test was performed to test the goodness of fit of the multivariable model. A Bonferroni-corrected *p*-value of <0.05 was considered statistically significant. Stata 15 [12]/R studio Version 1.2.1335 [13] was used for statistical analysis.

## 3. Results

### 3.1. Cohort Characteristics and Factors Associated with the Utilization of APM

During the study period, 10,769 patients were admitted to our institution’s neurocritical care service. Of these, 2258 patients (mean age 56.7 ± 17.8 years, 39.8% female (n = 899) received serial APM assessments within the first seven days of ICU admission. The median admission GCS was 6 (interquartile range, 3–15). During the ICU stay, NPi assessments were carried out for a median of 2.9 days (IQR, 0.77; 10.5). In total, 38,162 NPi observations were generated during the study period, with a median NPi observation count of 7 and an interquartile range of 3–16, with a maximum of 436 observations for one patient. Overall, 88.5% (n = 1998) were mechanically ventilated, while 46.7% (n = 1054) patients received intracranial pressure monitoring and 2.9% (n = 969) patients underwent electroencephalography, and 30.2% (n = 683) received hyperosmolar therapy. The average ICU length of stay (LOS) was 10.0 ± 10.7 days, and the average hospital LOS was 24.6 ± 29.5 days. Overall, 11.5% (n = 259) received a tracheostomy during their ICU admission, while 27.5% (n = 621) were transitioned to comfort measures only. At the time of discharge, 59.4% (n = 1341) were either dead (38.4%, n = 865) or had transitioned to a hospice (0.04%, n = 11) or a skilled nursing facility (20.6%, n = 465), while 40.6% (n = 917) were either discharged to home (22.6%, n = 348) or a rehabilitation facility (25.5%, n = 568). The detailed characteristics of patients receiving serial APM assessments are presented in Table 1.

### 3.2. The Temporal Trends in NPi Values

Out of the 2258 patients with serial APM testing, 47.7% (n = 1078) exhibited NPi > 3 on both initial and final assessments, and 19% (n = 430) had initial NPi ≥ 3, but the NPi worsened to <3 subsequently without recovering. Another 30.1% (n = 680) had an initial NPI < 3 that never improved, and 3.1% (n = 70) had an initial NPi < 3, which did subsequently improve to >3. These trends were observed across different ABI etiologies and are shown in Table 2. However, the median NPi assessment period differed by the individual patients’ NPi temporal trends. The median NPi period was longest in patients with worsening NPi assessments and shortest in patients with improving assessments, i.e., in patients with NPi initially ≥3 that worsened to <3; the median days of NPi recordings were 10.1 (IQR 3.3; 19.2 days) and 6.5 (IQR 1.4; 16.4 days) in patients with NPi < 3 that never improved, 1.6 (0.5; 5.9 days) in patients with stable NPi, and 1.1 (IQR 0.3; 4 days) in patients with NPi < 3 that subsequently improved. This trend was statistically significant (*p* < 0.001). Among the patients with an admission GCS score of 13–15 (n = 507), NPi worsened in 84 (16.6%). In patients with an admission GCS score 9–12 (n = 295), NPi worsened in 48 (16.3%), while in patients with an admission GCS score of 3–8 (n = 1456), NPi worsened in 298 (20.5%). 

### 3.3. Primary Outcome

The association between NPi trajectory and discharge disposition was investigated using a multivariable logistic regression model adjusted for age, sex, admission diagnosis, admission GCS, craniotomy/craniectomy, and hyperosmolar therapy. Patients with an NPi < 3 that did not recover (aOR 2.58, 95% CI (2.03, 3.28], *p* < 0.001) and patients with NPi ≥ 3 that subsequently worsened to <3 (aOR 2.09, 95% CI [1.61, 2.73], *p* < 0.001) were more likely to be associated with poor discharge outcomes, as shown in Table 3.

### 3.4. Secondary Outcomes

Patients with NPi > 3 that subsequently worsened to <3 (aOR 1.58, 95% CI [1.13, 2.22], *p* = 0.008) were more likely to receive tracheotomy tube placement. Patients with NPi < 3 that never improved (aOR 2.12, 95% CI [1.67, 2.70], *p* < 0.0001) and NPi > 3 that subsequently worsened to NPi < 3 (aOR 2.00, 95% CI [1.52, 2.68], *p* < 0.0001) were more likely to be associated with a transition to comfort measures. Table 3 describes the association between NPi trajectory and clinical outcomes. The Hosmer–Lemeshow goodness of fit test demonstrated good model fit for all three outcomes of interest. 

## 4. Discussion

In this retrospective observational study of NPi trends among 2258 patients admitted to the neurocritical care unit with acute brain injury, one-third exhibited negative (worsening) NPi trajectory over time or initially low NPi without improvement. This is noteworthy because of the statistically significant association between unfavorable outcomes at hospital discharge and patients with persistently low or worsening NPi measurements. These results highlight the importance of monitoring NPi trends over time. It is yet to be determined if more aggressive interventions in patients who do not demonstrate early improvement in NPi or exhibit worsening NPi will improve outcomes. However, in the climate of precision-based individualized ICU care using high-frequency physiological monitoring, NPi plays a role. Overall, the study provides a valuable contribution to the field of neurocritical care by emphasizing the significance of NPi assessment as a prognostic tool and emphasizing the need for further research in this area.

That one-third of patients (30.1%) had an initial NPi < 3, which never improved, and 19% had initial NPi that subsequently worsened to <3 and never recovered is an important and striking observation. These patients may represent a subgroup of ABI patients with a particularly poor prognosis and thus may require closer monitoring and more aggressive management. Only a small percentage (3.1%) of patients had an initial NPi < 3 that subsequently improved. Perhaps, early intervention and aggressive management in these patients improved pupillary reactivity and subsequently improved outcomes. Further quantifications of persistently poor and worsening pupillary responses among this vulnerable patient population are necessary. This highlights the importance of continuous monitoring and suggests that early intervention in patients with ABI, particularly those with initial NPi < 3, may improve outcomes. Further quantifying persistently poor and worsening pupillary responses among this vulnerable patient population alongside detailed measures of various interventions will inform the development of targeted interventions to improve pupillary reactivity and ultimately improve outcomes in ABI patients.

Overall, the association between NPi trends and discharge outcomes suggests that NPi can be a valuable prognostic tool for providing intensive care to patients with ABI. The ability to predict poor outcomes at an early stage can help guide the management of patients with ABI, including the timing of interventions such as decompressive craniectomy or the use of medical therapies to optimize cerebral perfusion. The significant association between poor outcomes and patients with persistently low NPi < 3 is unsurprising. However, sluggish pupil reactivity is difficult to detect without APM with a pen light or flashlight and may be under-reported. Therefore, this study highlights the importance of the early identification of at-risk patients using high-fidelity pupillometers to hopefully facilitate timely interventions. Moreover, the association between poor outcomes and patients with NPi ≥ 3 who subsequently worsened emphasizes the need for increased vigilance, even when initial pupillary assessments are reassuring.

The finding that NPi trends were associated with the placement of a tracheostomy tube or the transition to comfort measures is important because it suggests that patients with NPi values < 3 may be perceived to be at risk of poor outcomes. Future studies examining time to tracheostomy in relation to NPi findings may shed more light on this association. The self-fulfilling prophecy, as it is termed and debated in many clinical scenarios, does not exclude the present cohort and must be noted when considering the retrospective findings of this study, especially with tracheostomy placement and transition to comfort measures. Tracheostomy placement, in particular, is a procedure that is often performed in critically ill patients with ABIs to provide long-term ventilator support. Because it is associated with substantial risks and long-term implications, identifying patients using trends in Npi who may benefit from the tracheostomy as a bridging intervention on the path to recovery may assist in making important medical and surgical decisions. However, we cannot exclude the possibility that NPi values may have led to medical and surgical interventions that could have benefited some patients. 

Similarly, the association between NPi trends (initial < 3 or NPi ≥ 3 that worsened) and the transition to comfort measures only emphasizes the importance of monitoring pupillary reactivity in patients with ABI. This finding suggests that neurocritical care providers may perceive patients with NPi < 3 to have a poor prognosis and might be less likely to recover from their injuries, leading to discussions with families and care teams about end-of-life care options. These findings highlight the potential clinical utility of monitoring NPi trends in patients with ABIs, particularly regarding informed medical interventions and end-of-life care decisions.

### Strengths and Limitations of the Study

Several limitations to our study should be considered when interpreting the results. First, this was a retrospective single-center study; therefore, the findings may not be generalizable to other institutions or populations. Even though we did not evaluate the immediate impact of specific interventions, such as surgery or medical management, on NPi trends and outcomes, our study results indicate that the NPi trend groups differed in relation to diagnostic and therapeutic intensity. Patients with NPI ≥ 3, which subsequently worsened to <3 demonstrated the highest proportion of ICP monitoring, the total number of hours of ICP monitoring, craniotomy/craniectomy, hyperosmolar therapy, and brief or continuous electroencephalography. Finally, the lack of standardization in the timing and frequency of NPi assessments may have affected the accuracy and reliability of our findings.

Some potential strengths of this study include the large sample size of patients collected over eight years with ABIs who received serial NPi assessments and the focus on examining temporal trends in NPi and their association with discharge outcomes. This study uniquely evaluated the association between NPi and discharge outcomes in patients with acute brain injury across different etiologies, including acute ischemic stroke, spontaneous intracerebral hemorrhage, aneurysmal subarachnoid hemorrhage, and traumatic brain injury. The study is the first to report on the association between NPi and transition to comfort measures, an important clinical outcome for patients with ABI. The study adds to the limited literature on using NPi trends in neuro-prognostication and may provide valuable information for clinical decision-making in neurocritical care settings.

## 5. Conclusions

In conclusion, our study demonstrates that temporal trends in the neurological pupillary index (NPi) are associated with hospital discharge disposition in patients admitted to the ICU with acute brain injury. Our findings suggest that serial NPi assessments may be helpful in predicting outcomes and guiding clinical decision-making in patients with ABI. Further studies are warranted to evaluate the potential benefit of interventions to improve NPi trends in this population.

## Figures and Tables

**Table 1:** Characteristics of neurocritical care unit patients who received serial automated pupillometer assessments.

	Overall (N = 2258)
Age in years, Mean (SD)	56.7 (17.8)
Sex	
Female	899 (39.8%)
Male	1359 (60.2%)
Diagnosis	
Acute ischemic stroke	918 (40.7%)
Spontaneous intracerebral hemorrhage	486 (21.5%)
Subarachnoid hemorrhage	430 (19.0%)
Traumatic brain injury	424 (18.8%)
Admission Glasgow Coma Score, GCS Median [IQR]	6 [3, 15]
GCS 13–15 (Mild)	507 (22.5%)
GCS 9–12 (Moderate)	295 (13.1%)
GCS 3–8 (Severe)	1456 (64.5%)
Mechanically Ventilated	1998 (88.5%)
Intracranial Pressure Monitoring	1054 (46.7%)
External ventricular drain	579 (25.6%)
Parenchymal/subdural	284 (12.6%)
Combination of EVD and parenchymal/subdural	191 (8.5%)
Total Intracranial Pressure Monitoring (hours), Mean (SD)	248 (265)
Electroencephalography	969 (42.9%)
Brief	566 (25.1%)
Continuous	710 (31.4%)
Craniotomy/Craniectomy	914 (40.5%)
Hyperosmolar Therapy (23.4% saline/mannitol)	683 (30.2%)
NPi Trajectory	
Initial NPi ≥ 3 or greater that remained ≥3	1078 (47.7%)
Initial NPi < 3, but subsequently improved to ≥3	70 (3.1%)
Initial NPi < 3, and remained <3,	680 (30.1%)
Initial NPi > 3, which subsequently worsened to <3 and did not recover	430 (19.0%)
Intensive Care Unit Length of Stay in Days, Mean (SD)	10.0 (10.7)
Hospital Length of Stay in Days, Mean (SD)	24.6 (29.5)
Tracheostomy Tube Placement	259 (11.5%)
Transition to Comfort Measures Only	621 (27.5%)
Discharge disposition	
Death	865 (38.4%)
Hospice	11 (0.04%)
Skilled nursing facility	465 (20.6%)
Home	348 (22.6%)
Rehabilitation facility	568 (25.5%)

**Table 2:** Characteristics of patients with temporal trends in NPi values.

	Initial NPi ≥ 3 and Remained ≥3 (N = 1078)	Initial NPi < 3 and Improved to ≥3 (N = 70)	Initial NPi < 3, and Remained <3 (N = 680)	Initial NPi ≥ 3, Worsened to <3 (N = 430)	Overall (N = 2258)
Age in Years, Mean (SD)	58.9 (17.5)	56.3 (17.7)	55.8 (18.0)	53.0 (17.1)	56.7 (17.8)
Sex					
Female	396 (36.7%)	33 (47.1%)	295 (43.4%)	175 (40.7%)	899 (39.8%)
Male	682 (63.3%)	37 (52.9%)	385 (56.6%)	255 (59.3%)	1359 (60.2%)
Diagnosis					
Acute ischemic stroke	460 (42.7%)	30 (42.9%)	234 (34.4%)	194 (45.1%)	918 (40.7%)
Spontaneous Intracerebral Hemorrhage	218 (20.2%)	9 (12.9%)	172 (25.3%)	87 (20.2%)	486 (21.5%)
Subarachnoid hemorrhage	203 (18.8%)	17 (24.3%)	126 (18.5%)	84 (19.5%)	430 (19.0%)
Traumatic brain injury	197 (18.3%)	14 (20.0%)	148 (21.8%)	65 (15.1%)	424 (18.8%)
Admission Glasgow Coma Score, Mean (SD)	8.14 (4.43)	8.66 (4.65)	6.49 (4.35)	7.17 (4.27)	7.48 (4.44)
GCS 13–15 (Mild)	278 (25.8%)	22 (31.4%)	123 (18.1%)	84 (19.5%)	507 (22.5%)
GCS 9–12 (Moderate)	169 (15.7%)	14 (20.0%)	64 (9.4%)	48 (11.2%)	295 (13.1%)
GCS 3–8 (Severe)	631 (58.5%)	34 (48.6%)	493 (72.5%)	298 (69.3%)	1456 (64.5%)
Mechanical Ventilation	922 (85.5%)	54 (77.1%)	611 (89.9%)	411 (95.6%)	1998 (88.5%)
Intracranial Pressure Monitoring	484 (44.9%)	30 (42.9%)	293 (43.1%)	247 (57.4%)	1054 (46.7%)
External ventricular drain	259 (24.0%)	15 (21.4%)	187 (27.5%)	118 (27.4%)	579 (25.6%)
Parenchymal/subdural	138 (12.8%)	7 (10.0%)	68 (10.0%)	71 (16.5%)	284 (12.6%)
Combination of EVD and parenchymal/subdural	87 (8.1%)	8 (11.4%)	38 (5.6%)	58 (13.5%)	191 (8.5%)
Intracranial Pressure Monitoring Period in Hours, mean (SD)	251 (250)	224 (205)	215 (244)	282 (314)	248 (265)
Electroencephalography					
Brief	282 (26.2%)	14 (20.0%)	130 (19.1%)	140 (32.6%)	566 (25.1%)
Continuous	331 (30.7%)	17 (24.3%)	165 (24.3%)	197 (45.8%)	710 (31.4%)
Craniotomy/Craniectomy	436 (40.4%)	28 (40.0%)	230 (33.8%)	220 (51.2%)	914 (40.5%)
Hyperosmolar Therapy	219 (20.3%)	16 (22.9%)	262 (38.5%)	192 (44.6%)	680 (30.1%)
Intensive Care Unit Length of Stay in Days, Mean (SD)	10.2 (10.0)	8.09 (6.72)	8.21 (10.8)	12.8 (11.9)	10.0 (10.7)
Hospital Length of Stay in Days, Mean (SD)	26.4 (26.7)	24.5 (23.6)	18.0 (29.5)	30.3 (34.8)	24.6 (29.5)
Tracheostomy Tube Placement	110 (10.2%)	4 (5.7%)	64 (9.4%)	81 (18.8%)	259 (11.5%)
Transition to Comfort Measures Only	217 (20.1%)	14 (20.0%)	253 (37.2%)	137 (31.9%)	621 (27.5%)
Discharge Disposition					
Home/rehabilitation facility	519 (48.1%)	37 (52.9%)	207 (30.4%)	154 (35.8%)	917 (40.6%)
Death/hospice/skilled nursing facility	559 (51.9%)	33 (47.1%)	473 (69.6%)	276 (64.2%)	1341 (59.4%)

**Table 3:** The association between NPi trajectory and clinical outcomes.

Outcomes of Interest	Adjusted Odds Ratio	95% CI	*p*-Value *	Hosmer–Lemeshow Goodness of Fit Test *p*-Value
Death/Hospice/Skilled Nursing Facility				0.17
Initial NPi ≥ 3 or greater that remained ≥3	Reference group			
Initial NPi < 3, but subsequently improved to ≥3	0.89	0.53, 1.61	0.78	
Initial NPi < 3, and remained <3,	2.58	2.03, 3.28	<0.001	
Initial NPi > 3, which subsequently worsened to <3 and did not recover	2.09	1.61, 2.73	<0.001	
Model adjusted for age, sex, admitting diagnosis, admission Glasgow Coma Scale Score, mechanical ventilation, craniotomy/craniectomy, and hyperosmolar therapy.				
Tracheostomy				0.10
Initial NPi ≥ 3 or greater that remained ≥3	Reference group			
Initial NPi < 3, but subsequently improved to ≥3	0.55	0.19, 1.57	0.69	
Initial NPi < 3, and remained <3,	0.79	0.55, 1.12	0.27	
Initial NPi > 3, which subsequently worsened to <3 and did not recover	1.58	1.13, 2.22	0.008	
Model adjusted for age, sex, admitting diagnosis, admission Glasgow Coma Scale Score, craniotomy/craniectomy, and hyperosmolar therapy.				
Transition to Comfort Measures Only				0.98
Initial NPi ≥ 3 or greater that remained ≥3	Reference group			
Initial NPi < 3, but subsequently improved to ≥3	1.25	0.65, 2.40	0.50	
Initial NPi < 3, and remained <3,	2.12	1.67, 2.70	<0.001	
Initial NPi > 3, which subsequently worsened to <3 and did not recover	2.00	1.52, 2.68	<0.001	

Model adjusted for age, sex, admitting diagnosis, admission Glasgow Coma Scale score, mechanical ventilation, craniotomy/craniectomy, and hyperosmolar therapy. Abbreviations: CI, confidence interval. * Bonferroni-corrected.

## Data Availability

Data for this study are not publicly available.
